# Complicated jejunal diverticulosis with intestinal perforation and obstruction: delay in hospital visit during confinement due to COVID-19

**DOI:** 10.1093/jscr/rjac010

**Published:** 2022-02-11

**Authors:** María S Ponce Beti, René M Palacios Huatuco, Santiago Picco, Alejandro E Capra, Daniel G Perussia, Alejando M Suizer

**Affiliations:** Department of General Surgery, Hospital Militar Regional Córdoba, Av. Cruz Roja Argentina 1174, Córdoba Capital, Argentina; Department of General Surgery, Clínica Universitaria Reina Fabiola, Universidad Católica de Córdoba, Oncativo 1248, Córdoba Capital, Argentina; Department of General Surgery, Hospital Militar Regional Córdoba, Av. Cruz Roja Argentina 1174, Córdoba Capital, Argentina; Department of General Surgery, Hospital Militar Regional Córdoba, Av. Cruz Roja Argentina 1174, Córdoba Capital, Argentina; Department of General Surgery, Hospital Militar Regional Córdoba, Av. Cruz Roja Argentina 1174, Córdoba Capital, Argentina; Department of General Surgery, Hospital Militar Regional Córdoba, Av. Cruz Roja Argentina 1174, Córdoba Capital, Argentina

## Abstract

Diverticulosis of the small bowel is a rare entity. It can cause acute, complications such as diverticulitis, perforation, intestinal bleeding and obstruction. During the pandemic, patients were reluctant to visit hospitals for fear of contracting coronavirus disease 2019. This caused the patients to wait until the extreme deterioration of many acute surgical conditions. An 83-year-old man with multiple comorbidities showed up at the emergency department with generalized abdominal pain of 7 days of evolution. The computed tomography scan revealed a large distention of the small intestine and a small inflammatory abscess. He was transferred to the operating room where a segment of the jejunum affected by multiple diverticula located on the mesenteric side of the intestine and a mesenteric abscess related to a perforated jejunal diverticulum were identified. Complicated jejunal diverticulosis is a difficult entity to diagnose, which can cause significant morbidity and mortality. To avoid this, its timely diagnosis is essential.

## INTRODUCTION

Small intestinal diverticula are very rare, with an incidence of 0.3–2.3% [1]. The prevalence of diverticula in the proximal jejunum, distal jejunum, and ileum is 75%, 20% and 5%, respectively [2]. Although most cases are asymptomatic, 30–40% may present with chronic abdominal pain, malabsorption, hemorrhage, diverticulitis, obstruction, abscess formation and perforation [3].

We present the case of an 83-year-old man who, due to his comorbidities and confinement during the coronavirus disease 2019 (COVID-19) pandemic, consulted late for jejunal diverticulosis and various complications.

## CASE REPORT

An 83-year-old man was admitted to the emergency department with colicky abdominal pain for 7 days. His medical history was significant for arterial hypertension, benign prostatic hyperplasia and chronic obstructive pulmonary disease, with no previous abdominal surgeries. He reported that the pain started in the periumbilical region and it was subsequently intensified in a generalized way. It was associated with hyporexia, constipation and poor gas elimination. He denied other symptoms.

On admission, the patient had a pulse of 89 bpm, blood pressure of 120/70 mm Hg, and temperature of 36.5°C. On physical examination he revealed abdominal distention, hyperactive bowel sounds on auscultation, tympanic sound on percussion and mild tenderness on palpation. No hernia was noted and no palpable mass was identified.

Blood tests showed leukocytosis 13.2 × 10^9^/L with 88% segmented neutrophils, C-reactive protein 5 mg/L and hemoglobin of 12.5 g/dl. Metabolic panel and liver function tests were within normal limits.

**Figure 1:**
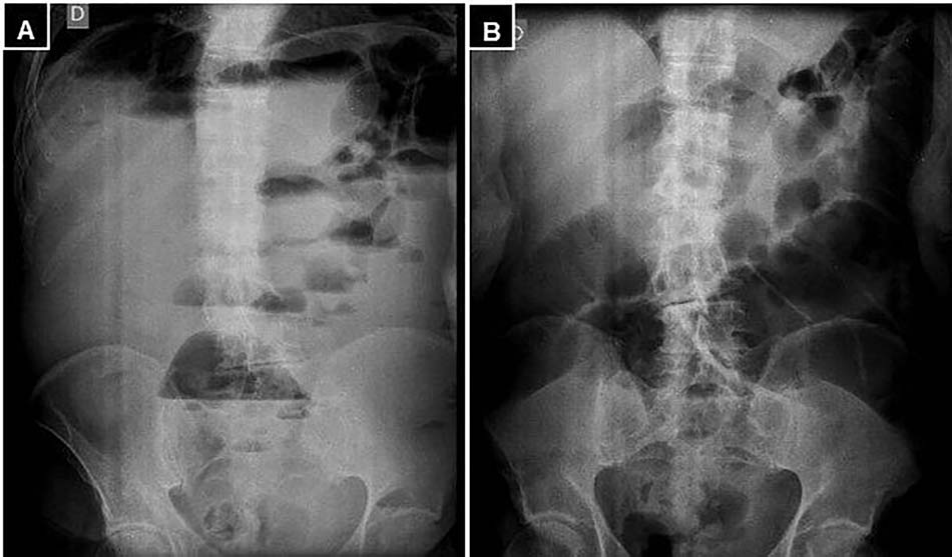
(**A)** Standing abdominal X-ray, and (**B)** supine position demonstrating marked distention of the small intestine and multiple air-fluid levels.

**Figure 2:**
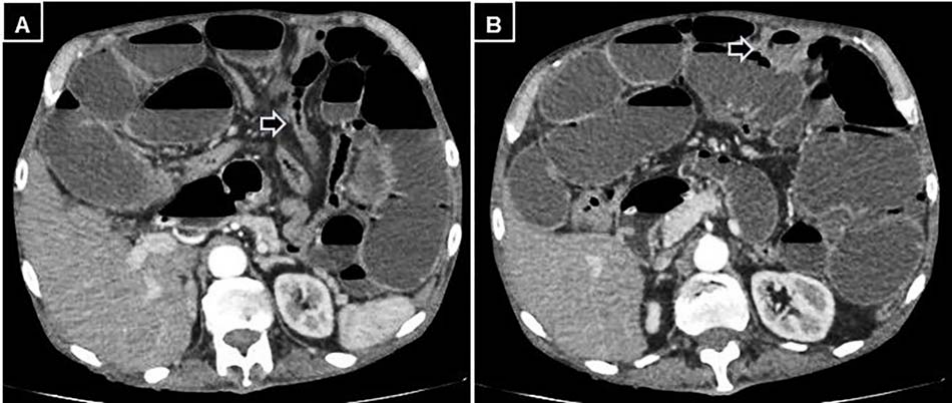
Abdominal and pelvic CT with intravenous contrast. (**A)** Axial image illustrating a change in caliber (black arrow) with subsequent distension of the small intestine, and (**B)** a small mesenteric collection (black arrow).

**Figure 3:**
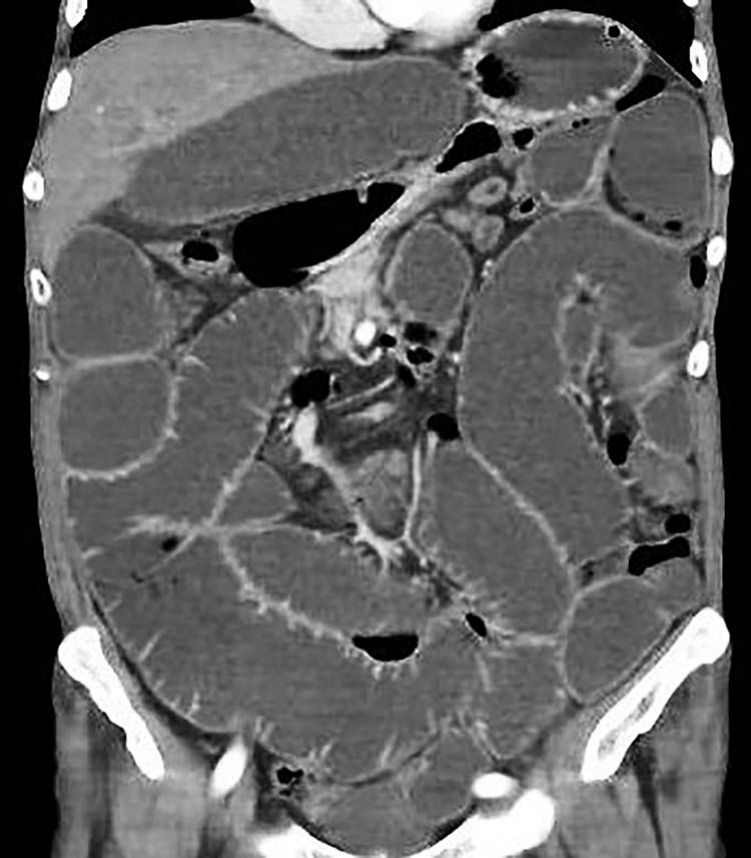
Abdominal and pelvic CT with intravenous contrast. Coronal image illustrating dilated fluid-filled loops of the small intestine consistent with high-grade mechanical obstruction.

A supine and upright plain X-ray of the abdomen were obtained and showed dilated small bowel loops, mainly in the left upper abdomen, along with multiple air-fluid levels (Fig. 1). Computed tomography (CT) demonstrated significant air-fluid distention of the entire small bowel, up to the right flank and periumbilical region, where a change in caliber was observed (Fig. 2). The findings corresponded to small bowel obstruction (Fig. 3).

**Figure 4:**
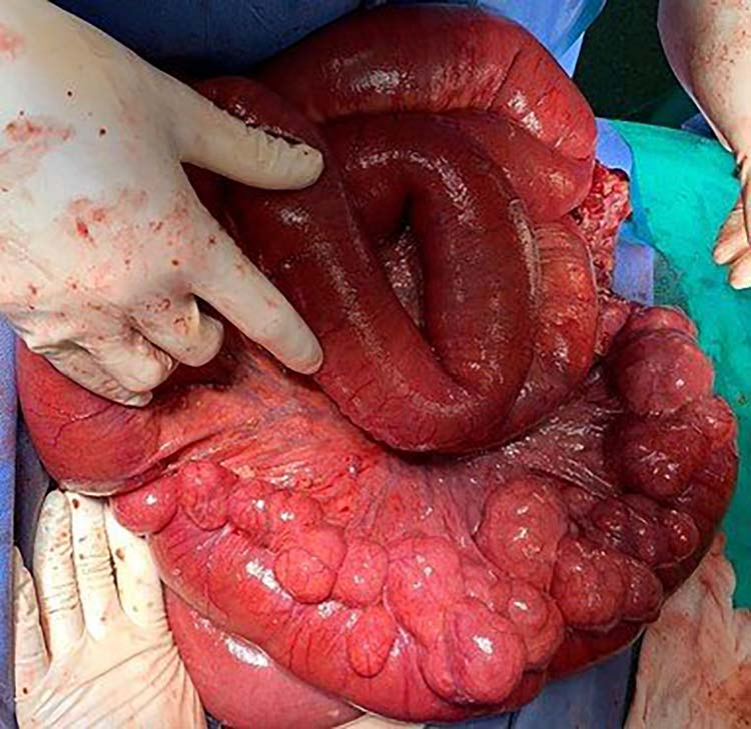
Jejunum segment affected by multiple diverticula located on the mesenteric side of the intestine.

**Figure 5:**
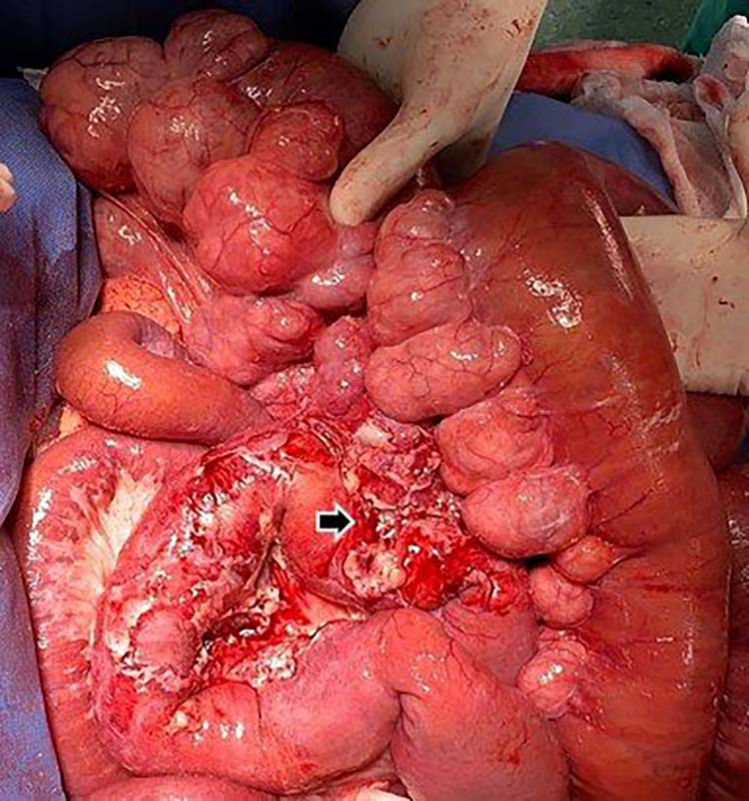
Inflammation of the peridiverticular mesenteric tissue adjacent to the perforated jejunal diverticulum (black arrow).

It was decided to perform an exploratory laparotomy, recognizing complete distention of the small intestine and multiple diverticula in the jejunum and ileum (Fig. 4). The abdominal cavity was explored detecting an abscessed intestinal plastron (Fig. 5). A perforated diverticulum was recognized in the jejunum segment 160 cm after the Treitz Ligament. Segmental resection of 10 cm of the small bowel was performed. However, the patient presented hemodynamic instability; therefore, only an ileostomy associated with an open and contained abdomen was performed. The operative time was 60 min, and the amount of blood loss was 750 ml. Two units of red blood cells were required.

The immediate postoperative follow-up was carried out in the intensive care unit. However, the patient died 6 h later.

**Figure 6:**
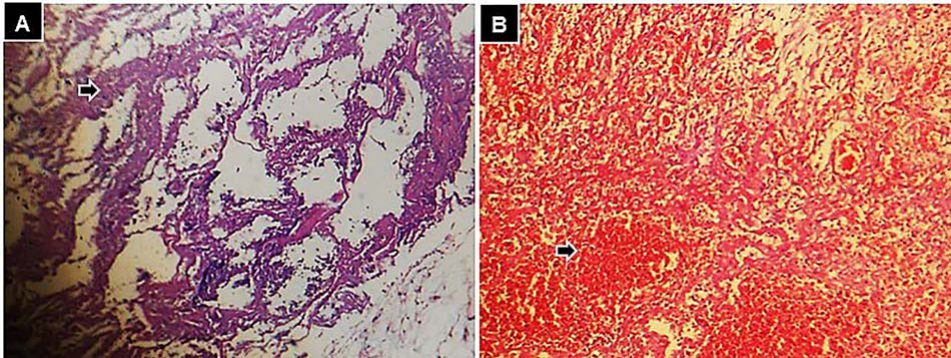
Histological findings following hematoxylin and eosin staining, 10× small bowel, (**A)** zone of transmural necrosis (black arrow), and (**B)** intestinal wall with zones of necrosis, and hemorrhage (black arrow).

The histopathological study reported transmural necrosis and acute abscess peritonitis (Fig. 6).

## DISCUSSION

Jejunal diverticular disease is a rare entity, which ranks last in frequency within diverticular disease [4]. In the small intestine, most diverticula are located in the proximal jejunum, close to the ligament of Treitz [2]. They are characterized by herniation of the mucosa and submucosa through the muscle layer at the point where blood vessels enter the intestinal wall [3]. This explains its typical location on the mesenteric side of the intestine [5]. They affect males in a higher proportion (2: 1), with a peak incidence between the sixth and seventh decades of life [6]. These findings were similar in our patient.

Clinical suspicion of jejunal diverticulosis and its complications remains difficult, as its diagnosis is often missed or delayed in relation to its nonspecific symptoms of acute abdominal pain with infectious syndrome and rarely gastrointestinal bleeding [7].

The diagnosis of small bowel diverticular disease is largely made by imaging [8]. Currently, diagnosis is typically made on the basis of abdominal CT scan or magnetic resonance imaging [9]. In an acute presentation, CT finding includes of bowel wall thickening with smooth margins, mesenteric edema and inflammation, extraluminal free air and fluid collection around diverticula [8]. However, interpretation of CT images requires an experienced radiologist and some degree of clinical suspicion [9]. In our case, diverticulosis could not be identified preoperatively due to the great intestinal distension and the accumulation of intramural fluid.

The incidence of each complication is variable and difficult to assess due to the small number of cases in most studies. However, it seems that diverticulitis (2–6%) and perforation (2.1–7%) are the most frequent complications, followed by bleeding (2–8.1%) and obstruction (2.3–4.6%) [7, 10]. Diverticulitis can be complicated by perforation of the diverticulum, leading to localized or generalized peritonitis, but may more commonly cause a mesenteric abscess. In these cases, the defect of peritoneal contamination would delay the diagnosis and explain the disastrous clinical course in debilitated and/or elderly patients [11]. This coincides with the clinical presentation of our case. On the other hand, mechanical intestinal obstruction may be related to extrinsic intestinal compression by a pseudoinflammatory tumor due to jejunal diverticulitis [12]. This manifestation was what finally motivated our patient to make the consultation, but belatedly.

These complications are associated with a significant mortality rate and in many cases, this is due to a delay in diagnosis [13]. It has been reported that during the pandemic, patients were reluctant to visit hospitals for fear of contracting COVID. This caused the patients to wait until the extreme deterioration of many acute surgical conditions. Both factors resulted in increased morbidity and mortality [14, 15]. In this scenario, we believe that our patient delayed the consultation for 7 days due to social confinement and late clinical manifestations of contained and localized intestinal perforation, which had a significant impact on the stage of his disease and outcome.

When acute complications is appeared, surgical intervention is usually required [8]. The chosen technique will depend on the circumstances of each case; in general, intestinal resection of the affected segment and primary end-to-end anastomosis is recommended. In the case presented, segmental resection of the small intestine was performed, and anastomosis of the ends could not be performed due to hemodynamic instability of the patient.

In conclusion, complicated jejunal diverticulosis is a difficult entity to diagnose. When therapy is delayed, complications can have severe consequences. However, the confinement during the COVID-19 pandemic has caused patients to postpone medical consultation. In this scenario, to avoid the development of complications and thus avoid surgery, a high degree of clinical suspicion is needed so that diagnosis and treatment are timely. Therefore, this disease should be considered in the list of differential diagnoses of acute abdomen, especially in elderly patients.

## CONSENT FOR PUBLICATION

Informed consent for publication of their clinical details and/or clinical images was obtained from the patient. A copy of the consent form is available for review by the Editor of this journal.

## CONFLICT OF INTEREST STATEMENT

None declared.
